# Farnesyl diphosphate synthase is important for the maintenance of glioblastoma stemness

**DOI:** 10.1038/s12276-018-0166-2

**Published:** 2018-10-17

**Authors:** Hee Yeon Kim, Dong Keon Kim, Seung-Hyun Bae, HyeRan Gwak, Ji Hoon Jeon, Jong Kwang Kim, Byung Il Lee, Hye Jin You, Dong Hoon Shin, Young-Ho Kim, Soo Youl Kim, Sung-Sik Han, Jin-Kyoung Shim, Ji-Hyun Lee, Seok-Gu Kang, Hyonchol Jang

**Affiliations:** 1 Research Institute, National Cancer Center, Goyang, 10408 Republic of Korea; 2 Department of Cancer Biomedical Science, National Cancer Center Graduate School of Cancer Science and Policy, Goyang, 10408 Republic of Korea; 3 Department of Molecular Cell Biology, Sungkyunkwan University School of Medicine, Suwon, 16419 Republic of Korea; 4 Center for Liver Cancer, National Cancer Center, Goyang, 10408 Republic of Korea; 5 Department of Neurosurgery, Brain Tumor Center, Severance Hospital, Yonsei University College of Medicine, Seoul, 03722 Korea

## Abstract

Glioblastoma is a highly malignant tumor that easily acquires resistance to treatment. The stem-cell-like character (stemness) has been thought to be closely associated with the treatment resistance of glioblastoma cells. In this study, we determined that farnesyl diphosphate synthase (FDPS), a key enzyme in isoprenoid biosynthesis, plays an important role in maintaining glioblastoma stemness. A comparison of the mRNA expression in patient-derived glioblastoma sphere cells, which maintain stemness, and their differentiated counterparts, which lose stemness, via RNA sequencing showed that most of the altered genes were networked in the cholesterol biosynthesis pathway. We screened Federal Drug Administration (FDA)-approved drugs targeting specific enzymes in the cholesterol biosynthesis pathway for their ability to inhibit glioblastoma sphere formation. Inhibitors of FDPS, such as alendronate and zoledronate, significantly reduced the formation of glioblastoma spheres, and alendronate was effective at a lower molar concentration than zoledronate. Knockdown of FDPS using short hairpin RNA also completely inhibited the formation of secondary spheres. *FDPS* mRNA in patients with glioblastoma was associated with malignancy in three independent microarray data sets. RNA sequencing showed that alendronate treatment reduced the embryonic stem cell signature and activated development- and necrosis-related pathways in glioblastoma spheres. These results suggest that FDPS is important for the maintenance of glioblastoma stemness and that alendronate, a drug widely used to treat osteoporosis, can be repositioned to treat glioblastoma.

## Brain cancer: Enzyme target for potential therapy

A drug that targets a key enzyme in aggressive brain cancer tumors could help tackle resistance to existing treatments. Glioblastoma is the most aggressive form of brain cancer and remains difficult to treat because the cancer cells can survive chemotherapy and radiotherapy. Certain cells within glioblastoma tumors have ‘stemness’ – unique stem cell-like metabolic characteristics that allow them to rapidly repair DNA damage and trigger relapse. Hyonchol Jang at the National Cancer Center in Goyang, South Korea and co-workers discovered that an enzyme called farnesyl diphosphate synthase (FDPS) helps maintain stemness in glioblastoma. The team then treated patient-derived glioblastoma cells with existing drugs known to inhibit FDPS. One such drug, which is already used to treat osteoporosis, inhibited the formation of secondary glioblastoma and may prove valuble in the treatment of brain cancer.

## Introduction

Glioblastoma, which is the most common primary malignant brain tumor, had a low relative survival estimate of 5.5% at 5 years post-diagnosis in the United States in 2009–2013^1^. Glioblastoma is generally treated by surgery and a combination of radio- and chemotherapy. The current first-line chemotherapeutic drug for glioblastoma is temozolomide, which improves the median survival of patients by 2.5 months compared with radiotherapy alone^2,3^. The majority of the molecular targeted therapy trials for glioblastoma have not resulted in advances in survival^4^; thus, there is an urgent need to find novel candidates to treat glioblastoma.

Stem-cell-like properties (or stemness) has been considered one of the main reasons glioblastoma is refractory to treatment^5–7^. A small number of cancer cells within a heterogeneous cancer cell population exhibit stemness and can survive after therapeutic treatment^8,9^. Glioblastoma cells with stemness have an enhanced ability to repair damaged DNA and are more resistant to temozolomide compared with glioblastoma cells without stemness^10^. Thus, controlling stemness is important for effective treatment of patients with glioblastoma.

Cancer cells with stemness have a metabolism distinct from that of nearby non-stem cells in various cancers, including lung, ovarian, breast, and colon cancer^11–15^. Glioblastoma cells with stemness have altered oxygen consumption and lactate production compared with cells without stemness^16^; however, many issues remain unresolved. In this study, we found that the cholesterol biosynthetic-related pathways were specifically upregulated in patient-derived glioblastoma sphere cells, which were enriched in stemness, compared with their differentiated counterparts. In particular, farnesyl diphosphate synthase (FDPS), a key enzyme in isoprenoid biosynthesis, was found to play an important role in maintenance of glioblastoma stemness.

FDPS catalyzes the conversion of isopentenyl pyrophosphate and dimethylallyl pyrophosphate to geranyl pyrophosphate and farnesyl pyrophosphate, which are protein prenylation substrates. Because prenylation is important for many oncogenic proteins to exert their activity, prenylation inhibitors have been actively tested in clinical trials to treat various cancers^17,18^. FDPS has been implicated in glioblastoma drug resistance^19^, and the FDPS inhibitor zoledronate^20^ is used to treat bone metastasis^21,22^. These reports suggest that FDPS might be a potential target for cancer treatment. In this study, we found that FDPS was important for maintaining glioblastoma stemness. Moreover, the FDPS inhibitor alendronate^23^ significantly suppressed formation of glioblastoma spheres. Because alendronate has been approved by the Food and Drug Administration (FDA) and is widely used to treat osteoporosis^24,25^, our results suggest that alendronate could be repositioned to treat glioblastoma.

## Materials and methods

### Cell culture and chemicals

Patient-derived TS13-18 and TS13-20 cells were directly established from fresh male WHO grade 4 glioblastoma patient tissues in accordance with a protocol approved by the Institutional Review Board of Severance Hospital, Yonsei University College of Medicine (4-2012-0212).

We followed previously published methods to isolate tumor spheres (TSs) from the human brain^26^. These cells were cultured as TSs in DMEM/F-12 medium (#10-0900 cv, HyClone, Logan, UT, USA) supplemented with 1 × B27 (#17504-044, Invitrogen, San Diego, CA, USA), 20 ng/ml basic fibroblast growth factor (#E0291; Sigma-Aldrich, St. Louis, MO, USA), 20 ng/ml epidermal growth factor (#E9644, Sigma-Aldrich), and 1% penicillin-streptomycin (#15140-122, Invitrogen) at 37 °C in a 5% CO_2_ humidified incubator. The differentiated counterparts were cultured under the same conditions but supplemented with 10% heat-inactivated fetal bovine serum (FBS; #SH30084.03; HyClone). 293FT cells were maintained in DMEM supplemented with 1% penicillin-streptomycin (#15140-122; Invitrogen), Cellmaxin (#C3319-020; GenDEPOT, Austin, TX, USA), and 10% heat-inactivated FBS.

Lovastatin (mevinolin; #M2147), squalestatin 1 (zaragozic acid A; #Z2626), alendronate (alendronate sodium trihydrate; #A4978), zoledronate (zoledronic acid monohydrate; #SML0223), and staurosporine (#S5921) were purchased from Sigma-Aldrich. Hydrogen peroxide (#H1222) was obtained from Tokyo chemical industry.

### RNA sequencing

Total RNA from TS13-20 sphere cells was extracted with Trizol (#15596018, Life Technologies, Carlsbad, CA, USA) per the manufacturer’s instructions. Preparation of an RNA library and sequencing were performed by LAS (Seoul, Korea) in the case of TSs and their differentiated counterparts and by Macrogen (Seoul, Korea) in the case of alendronate-treated and non-treated TSs. Sequencing was performed using the Next sequencing 500 system and the HiSeq 2500 sequencing system (Illumina, San Diego, CA, USA).

### Analysis of RNA sequencing data

Gene set enrichment analysis was carried out with GSEA version 2.2.2. A *p*-value was determined for the mean rank of each gene set. Neural stem cell gene set data were obtained from Harmonizome (http://amp.pharm.mssm.edu/Harmonizome/)^27^. Embryonic stem cell gene set data were obtained from the GSEA Molecular signature database v6.0 (http://software.broadinstitute.org/gsea/index.jsp) by combining two gene sets: HATTACHARYZ_

EMBRYONIC_STEM_CELL and WONG_EMBRYONIC_STEM_CELL_CORE. The genes in each gene set are listed in Supplementary Table S1.

Functional enrichment analysis for biological pathway was carried out with FunRich version 2 (http://www.funrich.org)^28^. Functionally grouped Gene Ontology (GO) terms analysis was visualized by use of ClueGO v.2.3.3 run through Cytoscape v.3.5.1^29^ based on Kyoto Encyclopedia of Genes and Genomes (KEGG) (http://apps.cytoscape.org/apps/cluego).

RNA sequencing data from alendronate-treated and non-treated cells were analyzed via core analysis using Ingenuity Pathways Analysis (IPA; Ingenuity Systems, www.ingenutiy.com). Functional analysis determined the biological functions that were most significant to the data set (*P* < 0.05).

#### RT-PCR and Real-time qPCR

Preparation of RNA, reverse-transcription polymerase chain reaction (RT-PCR), and real-time quantitative PCR (real-time qPCR) were performed according to a modified version of a method described previously^30^. Briefly, total RNA was extracted with Trizol (#15596018, Life Technologies), and cDNA was synthesized using AMV Reverse Transcriptase (#2620 A, Takara Bio, Shiga, Japan). Real-time qPCR was performed with a FastStart Essential DNA Green Master Kit (#06402712001, Roche Diagnostics, Indianapolis, IN, USA) using a Real-time PCR LightCycler96 (Roche Diagnostics). The primers used are listed below.

**Table:** 

Gene	Forward (5′– 3′)	Reverse (5′– 3′)
*ACTB*	CAAGATCATTGCTCCTCCTG	GAAAGGGTGTAACGCAACTA
*HMGCR*	TCGGTGGCCTCTAGTGAGAT	TCACTGCTCAAAACATCCTCTTC
*HMGCS1*	CCTGGTAGTTGCAGGAGATATTG	ACAGAATAGCAGCGGTCTAATG
*FDPS*	GTGCTGACTGAGGATGAGATG	GCTCGATCAGGTTCAGGTAATAG
*FDFT1*	GAGAAGGATCGCCAGGTGCT	AGCCCAGCAACATAGTGGCA
*HES6*	ATTGCCCGGAGTGTCTGGAG	GAGGGAGGGAAGACCTGGGA
*DLL3*	GTCCGAGCTCGTCCGTA	GGACAGAATCGAGGAAGGGT
*FUT9*	TCCAGATTCACTGCTCTCCC	ATTCTAAATCGGTCCTAGGGGT
*GFAP*	TTGCAGACCTGACAGACGCT	GCCTCTCCAGGGACTCGTTC

#### Imaging of sphere cells

Images of sphere cells were obtained using a Cyation-3 cell imaging multimode microplate reader with a ×4 objective (Bio-Tek, Winooski, VT, USA). Images were analyzed using the ImageJ program. Colonies >10 μm in diameter were considered sphere cells.

#### Limiting Dilution Assay

Limiting dilution assays were performed according to a modified version of a method described previously^31^. Cells were cultured in TS conditions with the indicated concentration of alendronate for 7 days.

#### Western blotting

Western blotting was performed according to a modified version of a method described previously^32^. Cells were lysed with lysis buffer containing 20 mM Tris-Cl (pH 7.4), 150 mM NaCl, 1 mM EDTA, 1% (v/v) Triton X-100, and protease inhibitors (#p3100-010, GenDEPOT). Anti-β-actin antibody (#A2228) was purchased from Sigma, and anti-FDPS antibody (#ab153805) was obtained from Abcam (Cambridge, MA, USA). Images were obtained using a Fusion SL/SOLO imaging system (Vilber Lourmat, France).

#### Confocal microscopy

Confocal microscopy was performed according to a modified version of a method described previously^33^. Tumor sphere cells were seeded in a Lab Tek II 8-chamber (#155409; Thermo Scientific, Waltham, MA, USA) coated with Cell Tak (#354240; Corning, Corning, NY, USA). The sphere cells were fixed in 4% paraformaldehyde, permeabilized with 0.5% Triton X-100, and stained with anti-FDPS antibody (#ab153805; Abcam) and Alexa Fluor 488-conjugated anti-rabbit antibody (#A11008; Thermo Scientific). Nuclei were stained with DAPI (#268298; Calbiochem, San Diego, CA, USA). Images were obtained at ×20 at the Imaging Core (National Cancer Center) on a LSM510 META or LSM780 confocal microscope (Carl Zeiss, Jena, Germany). For determination of the type of cell death, TS cells were treated with various death-inducing reagents, stained with Hoechst (#H3570, Thermo, 10 μg/ml) and propidium iodide (#LS-02-100, Biobud, 0.3 μg/ml) and then imaged with the LSM780 confocal microscope. Z-stack orthogonal projection images were processed with ZEN 2012 analysis software (Carl Zeiss, Germany).

#### Lentiviral short hairpin RNA (shRNA)-mediated FDPS knockdown

Human FDPS targeting sequences were designed using the Broad Institute GPP web portal (http://www.broadinstitute.org/

rnai/public/gene/search). Target sequences were synthesized by Macrogen (Seoul, Korea), annealed, and inserted into the EcoRI and AgeI sites of a pLKO.1 puro vector (Plasmid #8453, Addgene, Cambridge, MA, USA). As controls, sh-scrambled (Plasmid #1864, Addgene) or empty vector were used.

**Table:** 

	Target sequence (5′– 3′)
sh-FDPS #1	CCAGCAGTGTTCTTGCAATAT
sh-FDPS #2	CCCAGAGATAGGAGATGCTAT
sh-scrambled	CCTAAGGTTAAGTCGCCCTCG

Lentivirus was produced according to a modified version of a method described previously^34^. Experiments were carried out in accordance with the National Cancer Center, Institutional Biosafety Committee-approved protocol (17-NCCIBC-008). Briefly, 293FT cells were co-transfected with shRNA vectors and the packaging vectors psPAX2 (plasmid #12260; Addgene) and pMD2.G (plasmid #12259, Addgene) using polyethylenimine (#24313; Polysciences, Inc., Warrington, PA, USA). Two days after transfection, the culture media were filtered using a Minisart Syringe Filter (0.45 μm, #16555; Sartorius, Bohemia, NY, USA), and the lentivirus was concentrated using plus PEG-it Virus precipitation solution 5× (#LV810A-1, SBI).

Glioblastoma TS cells were infected with lentiviral shRNAs in the presence of 0.8 μg/ml polybrene (#H9268; Sigma) for 6 h, and the media were exchanged with fresh complete media. Two days after the infection, the infected cells were selected with 1 μg/ml puromycin (#AMR-J593; Amresco, Solon, OH, USA) for 2 additional days. Selected glioblastoma TS cells were seeded in 96-well clear flat-bottom, ultralow attachment microplates (#3474; Corning) and maintained for 4 days, and then images were obtained. The same number of cells were reseeded in 96-well microplates for secondary sphere culture and maintained for 6 days.

#### Statistics

Statistical analysis was performed as previously reported^12^. The data are presented as the means ± standard deviation, and *P* values were calculated using a Student’s *t* test calculator. All the data are representative of at least three separate experiments.

## Results

### **Patient-derived glioblastoma sphere cells are enriched in the stem cell signature**

**Fig. 1:**
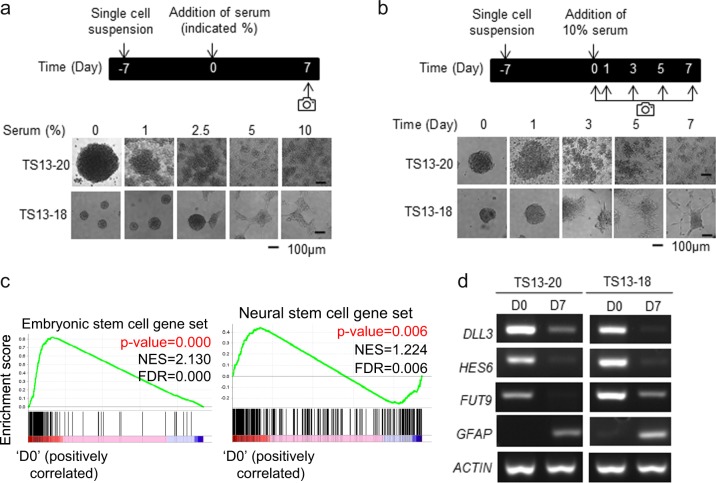
Patient-derived glioblastoma sphere cells are enriched in the stem cell signature. **a**, **b** Serum-induced differentiation of patient-derived glioblastoma sphere cells. Bright field image of cells taken using a Cytation 3 microplate reader (Bioteck) after the addition of (**a**) various concentrations of serum for 7 days and (**b**) 10% serum for the indicated number of days; a representative image of at least three independent experiments is shown. **c** The expression level of total RNA from TS13-20 spheres (D0) and their differentiated counterparts (D7) that were treated with 10% serum for 7 days was determined by RNA sequencing. Gene set enrichment analysis (GSEA) showed that sphere cells were enriched in neural stem cell and embryonic stem cell gene signatures. (NES: normalized enrichment score, FDR: false discovery rate). Gene sets are listed in Supplementary Table S1. **d** RNA sequencing results were validated by analyzing the expression of genes using reverse-transcription polymerase chain reaction (RT-PCR). *ACTB* was used as the loading control. Representative images of the 3 independent experiments are shown

First, we optimized the conditions to differentiate patient-derived glioblastoma sphere cells, which have been reported to harbor stemness^26,35,36^. Morphological differentiation was induced by adding 10% fetal bovine serum for 7 days to tumor sphere (TS) cultures of two independent patient-derived glioblastoma sphere cell sets, called TS13-20 and TS13-18 (Fig. 1a, b). Previously, we determined that glioblastoma patient-derived TS cells formed diffuse tumors that resembled that of the patient in a mouse orthotopic model and differentiated into astrocytes, oligodendrocytes, and the neuronal lineage^26^. We determined whether our TS cells harbored similarities to stem cells at the global mRNA level. Total RNA was isolated from TS13-20 sphere cells (marked D0) and their differentiated counterparts (marked D7, which were treated with 10% serum for 7 days) and analyzed via RNA sequencing. Gene set enrichment analysis (GSEA) showed that the TS cells were enriched in embryonic stem cell and neural stem cell signatures compared with their differentiated counterparts (Fig. 1c, Supplementary Table S1). The RNA-sequencing results were validated by RT-PCR for some of the genes (Fig. 1d). These results suggest that patient-derived glioblastoma sphere cells were enriched in the stem cell signature and lost their stemness after serum-induced differentiation.

### Cholesterol pathway genes are the most differentially expressed between sphere and differentiated cells

**Fig. 2:**
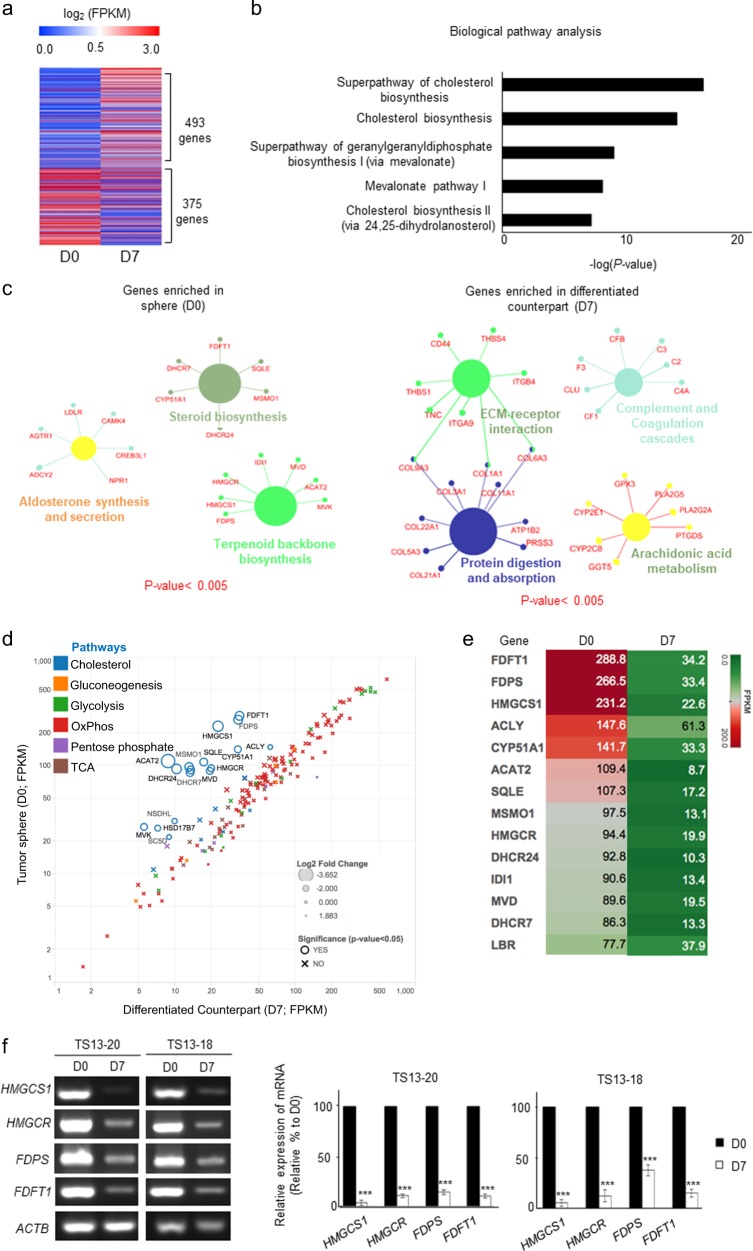
The most differentially expressed genes (DEGs) in sphere and differentiated cells are cholesterol pathway genes. **a** DEGs between TS13-20 spheres (D0) and their differentiated counterparts (D7) were analyzed by RNA sequencing. Genes with expression changes more than four-fold are shown. DEGs are listed in Supplementary Table S2. **b** The cholesterol biosynthesis-related pathway was the dominant pathway altered in sphere (D0) cells according to functional enrichment analysis using FunRich (http://www.funrich.org). The top five pathways are presented. **c** Down- and upregulated DEGs were subjected to gene network analysis using Cytoscape (http://www.cytoscape.org). Gene networks with *P* values < 0.005 are shown. **d** The change in the mRNA expression of various metabolic pathway genes between D0 and D7 cells was analyzed. Cholesterol-related genes were the only metabolic genes that varied between the two samples. The marker size is proportional to the log2-fold change (D0/D7). **e** Heatmap showing a clear distinction between the cholesterol pathway-related gene expression levels in the two samples. **f** Changes in the expression of cholesterol pathway genes were validated by RT-PCR and real-time quantitative PCR. The data are presented as the means of three independent experiments. ***P < 0.001 relative to D0

We identified differentially expressed genes (DEGs) between TS13-20 sphere (D0) cells and their differentiated counterparts (D7). In total, 493 genes were upregulated and 375 genes were downregulated in D7 cells, which was more than four-fold that in D0 cells (Fig. 2a and Supplementary Table S2). A biological pathway analysis showed that cholesterol-related pathways were predominantly altered between sphere cells and their differentiated counterparts (Fig. 2b). Upregulated genes in sphere cells were categorized into steroid biosynthesis, aldosterone synthesis and secretion, and terpenoid backbone biosynthesis pathways, all of which are components of cholesterol biosynthesis (Fig. 2c left). Downregulated genes in sphere cells were categorized into extracellular matrix-receptor interaction, protein digestion and absorption, complement and coagulation cascades, and arachidonic acid metabolism pathways (Fig. 2c right). This result suggests that cholesterol biosynthesis was upregulated in patient-derived glioblastoma sphere cells.

Next, we determined whether other metabolic pathways were altered during differentiation of glioblastoma sphere cells. Among the reported gene sets in various metabolic pathways^12^, the cholesterol gene set was the only one that changed significantly (Fig. 2d). The most expressed genes in D0 cells were FDFT1, FDPS, and HMGCS1 (Fig. 2e). RT-PCR and real-time qPCR validated the RNA-sequencing results (Fig. 2f). Moreover, TS13-18 cells also upregulated HMGCS1, HMGCR, FDPS, and FDFT1 in sphere cells compared with their differentiated counterparts (Fig. 2f). These results suggest that the cholesterol pathway was specifically upregulated in TS cells harboring stemness and that the cholesterol pathway might play a role in maintaining glioblastoma stemness.

### Pharmacological inhibition of FDPS suppresses formation of glioblastoma sphere cells

**Fig. 3:**
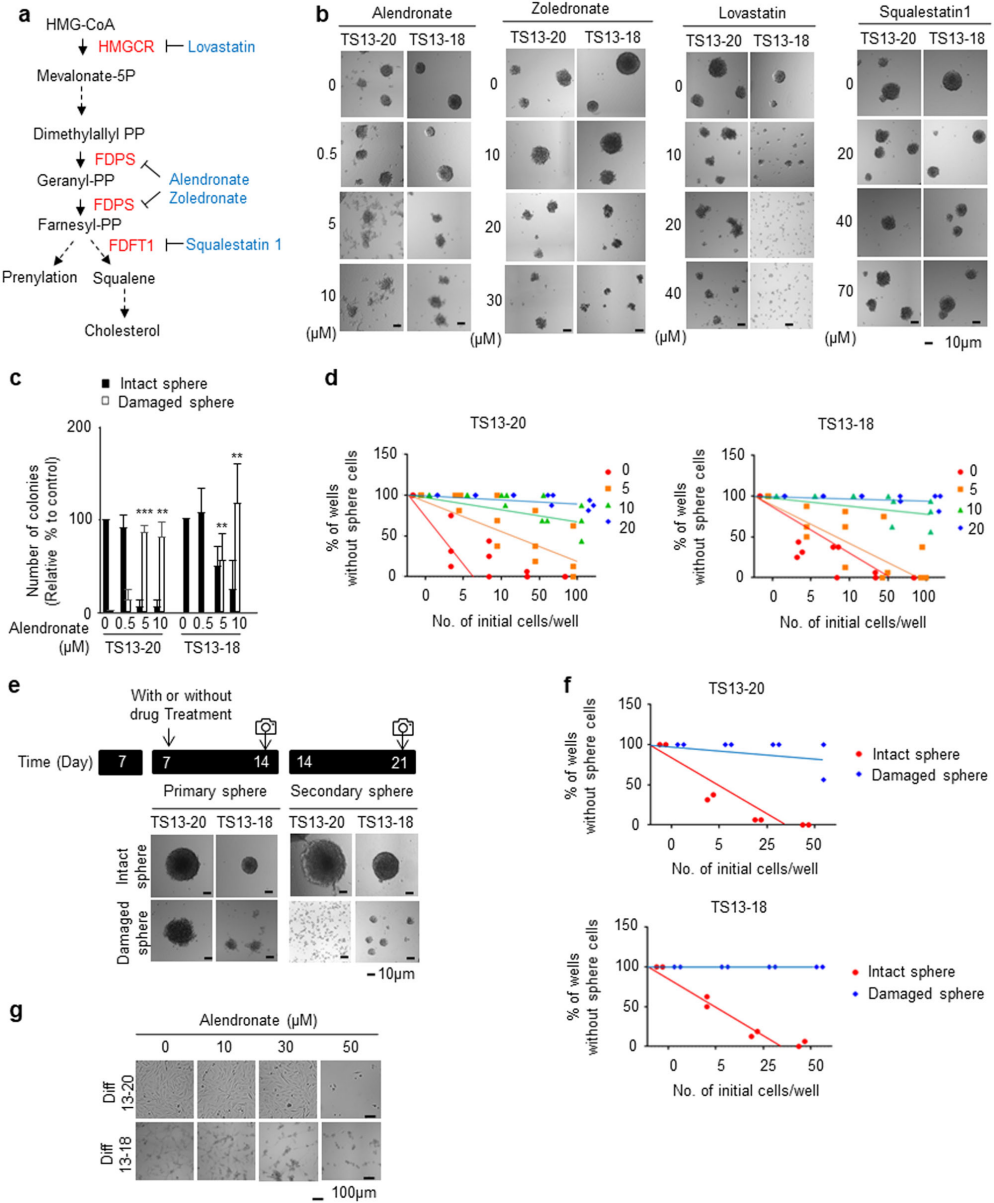
Pharmacological inhibition of farnesyl diphosphate synthase (FDPS) suppresses glioblastoma sphere formation. **a** Abbreviated representation of cholesterol synthesis. Black, metabolite; red, key enzymes; blue, FDA-approved drugs that inhibit the indicated enzymes. **b**, **c** Alendronate inhibited glioblastoma sphere formation. Patient-derived glioblastoma sphere cells (TS13-20 and TS13-18) were treated with the indicated concentrations of drugs for 7 days. Bright field images of cells were taken with a Cytation 3 microplate reader (Bio-Tek, Winooski, VT, USA). **b** A representative image of the experiment is shown. **c** Intact and damaged spheres were quantified (*n* = 3). ***P < 0.001, **P < 0.01, *P < 0.05, and NS *P* > 0.05 relative to the number of spheres cultured under no-drug-treatment conditions. **d** Limiting dilution assays were performed in TS13-20 and TS13-18 cells treated with the indicated concentration of alendronate. **e** Damaged spheres did not form secondary spheres. TSs cultured for 7 days were treated with or without alendronate for 7 days. Then, all sphere cells (intact and damaged) were subjected to secondary sphere formation. Bright field images of cells were taken 7 days after secondary culture using Cytation 3; a representative image of at least three independent experiments is shown. **f** Limiting dilution assays were performed with intact and damaged sphere cells under the same conditions described in (**e**). **g** Alendronate was less effective in differentiated cells than in sphere cells. Glioblastoma sphere cells (TS13-20, TS13-18) were differentiated for at least 7 days. Differentiated cells (Diff13-20, Diff13-18) were treated with the indicated concentration of alendronate for 7 days. Bright field images were obtained using a JuLI Br system (NanoEnTek, Inc., Korea); a representative image of at least three independent experiments is shown

To assess whether cholesterol biosynthesis is important for maintaining glioblastoma sphere cells, we treated the cells with pharmacological inhibitors of the core cholesterol pathway genes (Fig. 3a) during TS culture. Alendronate, zoledronate (FDA-approved FDPS inhibitors)^20,23,25^ and lovastatin (FDA-approved HMGCR inhibitor)^37^ apparently inhibited TS formation in patient-derived cells (TS13-20 and TS13-18) at 5–20 μM; however, squalestatin 1 (FDFT1 inhibitor)^38^ did not (Fig. 3b). Quantitative analysis of intact and damaged spheres showed that alendronate significantly decreased the number of intact spheres and significantly increased the number of damaged spheres at 5 μM for all TS cell types (Fig. 3c). Limiting dilution assays showed that alendronate reduced the stem cell population of TS cells at 5 μM for TS13-20 cells and at 10 μM for TS13-18 cells (Fig. 3d). Moreover, damaged spheres could not form TSs even in the absence of alendronate in the secondary TS formation assay (Fig. 3e). Quantitative analysis of the limiting dilution assays showed that 50 cells were sufficient to form secondary TSs in all the wells in a 96-well plate for intact TSs not treated with alendronate; however, alendronate-treated damaged TSs did not form secondary TSs in the majority of the wells (Fig. 3f). The differentiated counterparts of TS cells were relatively insensitive to alendronate, and their growth was inhibited at 30–50 μM (Fig. 3g). These results suggest that pharmacological inhibition of FDPS specifically suppressed the maintenance of glioblastoma stem cells.

### Knockdown of FDPS effectively inhibits tumor sphere formation

**Fig. 4:**
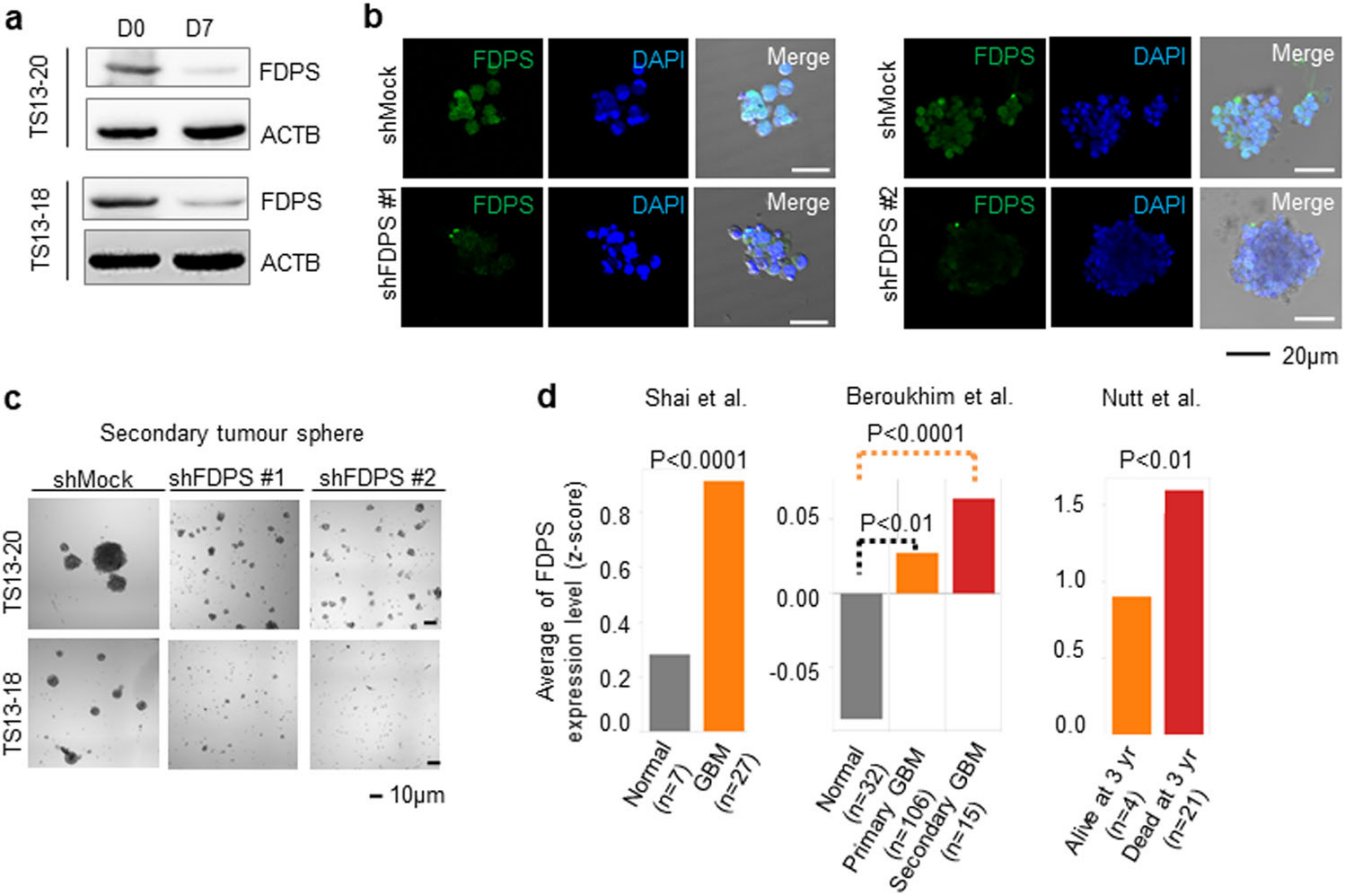
Knockdown of farnesyl diphosphate synthase (FDPS) suppresses glioblastoma sphere formation. **a** The protein levels of FDPS between TS13-20 and TS13-18 cell spheres (D0) and differentiated counterparts (D7) were analyzed by western blot. ACTB was used as the loading control. **b** TS13-20 cells were infected with lentivirus harboring short hairpin RNAs (shRNAs) against FDPS. Two days after infection, the infected cells were selected for 2 days and then maintained an additional 4 days. Knockdown of the FDPS protein was assessed by immunofluorescence staining and confocal microscopy. Images were obtained at ×20 using an LSM510 META or LSM780 confocal microscope (Carl Zeiss, Jena, Germany). **c** Knockdown of FDPS inhibited secondary sphere formation. The same number of control and FDPS knockdown cells were subjected to secondary sphere formation. Bright field images were obtained using a Cytation 3 microplate reader (Bio-Tek). **d** FDPS gene expression was significantly upregulated in glioblastoma cells compared with normal cells and in samples with a dead status at 3 years compared with samples with a live status. Three independent microarray-based data sets were median centered and normalized to the unit standard deviation to compare the gene expression level according to the *P*-value

We first checked whether the FDPS mRNA level was reflected by its protein level to confirm the role of FDPS in the formation of glioblastoma sphere cells. Western blotting showed that the FDPS protein expression level decreased significantly in differentiated cells compared with that in their TS counterparts (Fig. 4a). Then, we knocked down FDPS using short hairpin RNA (shRNA) and confirmed downregulation of the FDPS protein level via immunofluorescence staining and confocal microscopy (Fig. 4b). Knockdown of FDPS almost completely inhibited the formation of secondary TSs in patient-derived glioblastoma sphere cells (Fig. 4c), suggesting that FDPS is critical for the maintenance of glioblastoma sphere cells.

In accordance with these findings, FDPS mRNA was significantly upregulated in glioblastoma cells compared with normal cells (Fig. 4d left and middle) and in samples from patients who died within 3 years compared with samples from living patients (Fig. 4d right) in three independent microarray data sets^39–41^.

### Alendronate reduces the embryonic stem cell signature and activates the necrosis-related pathway

**Fig. 5:**
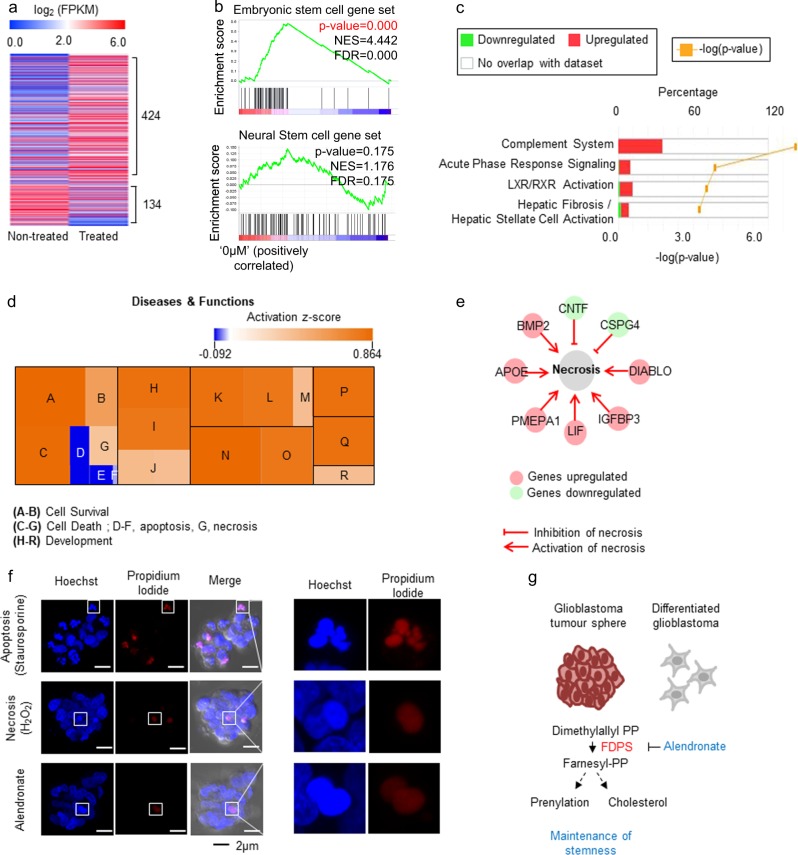
Alendronate reduces the embryonic stem cell signature and activates necrosis in glioblastoma spheres. **a** DEGs between TS13-20 cells treated with or without alendronate (10 μM) for 7 days were analyzed by RNA sequencing. Genes with expression changes greater than two-fold are shown. DEGs are listed in Supplementary Table S3. **b** GSEA showed that embryonic stem cell gene signatures were downregulated in alendronate-treated cells. **c**, **d** DEGs were analyzed by core analysis using Ingenuity® Pathway Analysis (IPA). **c** Canonical pathway analysis showed that the most functionally influenced mechanistic network was the complement system. **d** Downstream effect analysis showed that necrosis- and development-related genes were activated by treatment with alendronate in the disease and functions category. A: cell viability of neurons, B: cell viability, C: neuronal cell death, D–F: apoptosis, G: necrosis, H-J: cellular development, K-M: tissue development, N–O: nervous system development and function, P: embryonic development, Q: organismal development, R: connective tissue development and function. **e** The network of necrosis-related DEGs induced by treatment with alendronate is shown. **f** Alendronate caused necrosis-related cell death. TS13-20 cells were treated with staurosporine (0.5 μM, 18 h), hydrogen peroxide (1 mM. 18 h), or alendronate (10 μM, 3 days) and then stained with Hoechst and propidium iodide. Z-stack orthogonal projection images were obtained with an LSM780 confocal microscope and processed using ZEN 2012 analysis software (Carl Zeiss, Germany). **g** A model linking FDPS with glioblastoma stemness. Glioblastoma TS cells rely on FDPS for maintenance of stemness, and alendronate is a potential candidate drug for glioblastoma treatment

To investigate the molecular changes triggered by alendronate treatment, we analyzed DEGs between alendronate-treated and non-treated TS13-20 cells using RNA sequencing. In total, 424 genes were upregulated and 134 genes were downregulated in alendronate-treated cells, which was more than two-fold that in non-treated cells (Fig. 5a and Supplementary Table S3). GSEA showed that embryonic but not neural stem cell signatures were downregulated in alendronate-treated cells compared with non-treated cells (Fig. 5b). Core analysis by Ingenuity Pathway Analysis (IPA) showed that the most significantly altered canonical pathway was the complement system, which was upregulated by alendronate treatment (Fig. 5c). Downstream effect analysis of disease and functions showed that various developmental processes and necrosis were activated, whereas apoptosis was inactivated (Fig. 5d). Visualization of necrosis-related genes clearly showed that necrosis-promoting genes were upregulated and necrosis-inhibiting genes were downregulated by alendronate treatment (Fig. 5e). To validate these bioinformatic analysis results, we looked closely at the shape of the nuclei upon alendronate treatment. When assessing dead cells using propidium iodide, a membrane impermeable intercalating dye, alendronate-treatment-induced dead cells showed intact nuclei, which were similar to those in cells undergoing necrotic death induced by hydrogen peroxide treatment. Whereas cells undergoing apoptotic death caused by staurosporine showed fragmented nuclei^42^ (Fig. 5f). This result suggests that alendronate induces glioblastoma stem cell differentiation and causes cell death via the necrosis-related pathway. Overall, our findings suggest that glioblastoma cells rely on FDPS for the maintenance of stemness and that alendronate is a potential candidate drug for glioblastoma treatment (Fig. 5g).

## Discussion

Cancer cells maintain or acquire stemness by exploiting that of normal stem cells. Normal stem cells are generally classified as embryonic or adult stem cells. Distinct core transcriptional networks govern embryonic and adult stemness; embryonic stem cells rely on OCT4, SOX2, and NANOG for maintenance of pluripotency^43^, whereas the key players in adult cell stemness have not been fully revealed. A recent report showed that the core networks governing neural stem cells completely differ from those governing embryonic stem cells^44^. Thus, it is important to determine whether cancer exploits normal embryonic or adult stemness characteristics. Aggressive cancer cells are enriched in the embryonic stem cell-like gene expression signature^45^, and the embryonic stem cell-like transcriptional program strongly predicts metastasis and death^46^, suggesting that cancer cells exploit embryonic stemness. In contrast, two recent reports clearly show that lung cancer cells with stemness rely on normal lung adult stem-cell-specific signaling pathways^47,48^. In the present study, the GSEA showed that glioblastoma TS cells were enriched in both neural and embryonic stem cell genes (Fig. 1), suggesting that cancer can exploit both embryonic and adult stemness simultaneously.

We found that the cholesterol biosynthesis of glioblastoma cells with stemness distinctly differed from that of those without stemness (Fig. 2). The cholesterol pathway has been linked to glioblastoma; however, its link to glioblastoma stem cells has not been rigorously investigated. Upregulation of mevalonate and the cholesterol synthesis pathway is associated with poor survival in patients with glioblastoma^49^. HMGCR, a key cholesterol biosynthetic enzyme, has been widely studied as a target for glioblastoma treatment, and its inhibitors, statins, are under various clinical trials for glioblastoma treatment, although most trials are in the early stage (clinicaltrials.gov). Recent studies have shown that glioblastoma depends on cholesterol for survival and that it is sensitive to liver X receptor agonists^50^ or inhibition of sterol regulatory element-binding protein-1^51^. Considering that normal neural stem and progenitor cells are highly active in *de novo* lipid synthesis and depend on it for their proliferation^52^, glioblastoma cells might exploit normal neural stem cell metabolism for maintenance of their stemness.

FDPS has been implicated in the paclitaxel resistance of a glioblastoma cell line^19^, and Abate *et al*. showed that FDPS mRNA and protein levels, as well as enzyme activity, are upregulated in samples from patients with glioblastoma compared with normal human astrocytes and peripheral samples from tumor-free brains^53^. FDPS mRNA levels are also upregulated in glioblastoma samples compared with normal samples in other microarray data sets (Fig. 4d). Additionally, knockdown of FDPS almost completely blocks secondary tumor sphere formation by patient-derived glioblastoma tumor spheres (Fig. 4c). These results suggest that FDPS is a potential therapeutic target for glioblastoma treatment. Moreover, zoledronate and alendronate, which are FDA-approved FDPS inhibitors, have been widely used to treat osteoporosis. Zoledronate is undergoing clinical trials for treatment of various cancers, including prostate and breast cancer, but not glioblastoma (clinicaltrials.gov). Zoledronate enhances the anti-tumor effect of temozolomide in glioblastoma cell lines^54^; however, it does not cross the blood–brain barrier (BBB)^55^. In this study, alendronate inhibited the formation of glioblastoma spheres at a lower molar concentration than that of zoledronate (Fig. 3). Alendronate is currently not being evaluated in a clinical trial for glioblastoma treatment (clinicaltrials.gov); however, because it can cross the BBB^56^, our results suggest that it may be a good candidate for clinical trials aimed at glioblastoma treatment.

Recently, embryonic stemness was found to be important in the stemness index of human pan-cancer patients^57^. Alendronate reduced the embryonic stem cell signature in glioblastoma (Fig. 5b), and thus, it may also regulate stemness in various other cancers. Alendronate regulated not only stemness but also cell death. Although knockdown of FDPS resulted in apoptosis in glioblastoma cells cultured in adherent conditions^53^, our data suggest that alendronate triggers necrosis-related cell death in patient-derived glioblastoma TSs (Fig. 5d, f), reflecting differences between adherent and TS cultures of primary glioblastoma^35^. In addition, the complement system was upregulated by both differentiation induction and alendronate treatment in glioblastoma TSs (Figs. 2c and 5c). The complement system has been closely linked to cancer immunotherapy^58^, and thus, alendronate may be used in combination with anti-cancer immunotherapeutic drugs.

Farnesyl pyrophosphate, generated by FDPS, can be used to synthesize cholesterol via conversion to squalene by FDFT1, or it can be used in prenylation, which is a post-translational modification (Fig. 3a). In this study, inhibiting FDPS but not FDFT1 significantly affected TS formation (Fig. 3b), suggesting that protein prenylation may be important for glioblastoma TS formation. Indeed, abnormal protein prenylation has been attributed to the progression of several cancer types^17,18^, and prenylation of Ras and Rho has been implicated in glioma^59,60^. Thus, it is likely that FDPS affects TS maintenance by regulating protein prenylation.

Taken together, these data indicate that patient-derived glioblastoma sphere cells rely on the cholesterol biosynthesis-related pathway for maintenance of stemness. In particular, alendronate, an FDA-approved FDPS inhibitor, significantly suppressed maintenance of glioblastoma spheres. Our results suggest that an FDA-approved drug targeting cholesterol pathway-related genes could be repositioned for treatment of patients with glioblastoma.

## Electronic supplementary material

Supplemetary information legend

Supplementary Table S1

Supplementary Table S2

Supplemetary Table S3
