# Supplementary material for: Farnesyl diphosphate synthase is important for the maintenance of glioblastoma stemness
Source: Exp Mol Med. 2018 Oct 17;50(10):137. doi: 10.1038/s12276-018-0166-2 (PMC6193020; doi:10.1038/s12276-018-0166-2)
Supplement: Supplementary file 1 — Supplemetary information legend [file 12276_2018_166_MOESM1_ESM.pdf]

Supplementary Information

Farnesyl diphosphate synthase is important for the maintenance of glioblastoma stemness

Hee Yeon Kim[1,2], Dong Keon Kim[1,3], Seung-Hyun Bae[1], HyeRan Gwak[1], Ji Hoon Jeon[1], Jong Kwang Kim[1], Byung Il Lee[1,2], Hye Jin You[1,2], Dong Hoon Shin[1,2], Young-Ho Kim[1], Soo Youl Kim[1], Sung-Sik Han[4], Jin-Kyoung Shim[5], Ji-Hyun Lee[5], Seok-Gu Kang[5], and Hyonchol Jang[1,2,*]

Supplementary Tables

Supplementary Table 1 (related to Figure 1). Gene sets used in the gene set enrichment analysis for Figure 1c.

Supplementary Table 2 (related to Figure 2). Differentially expressed genes between TS13-20 tumor spheres and its differentiated counterpart.

Supplementary Table 3 (related to Figure 5). Differentially expressed genes between TS13-20 tumor spheres treated with or without alendronate.
